# Associations of family affluence with cortisol production and telomere length in European children

**DOI:** 10.1016/j.ebiom.2025.105793

**Published:** 2025-06-05

**Authors:** Kendal Marston, Chung-Ho E. Lau, Sandra Andrusaityte, Sunil Bhopal, Regina Grazuleviciene, Kristine Bjerve Gutzkow, Noemi Haro, Marianna Karachaliou, Katerina Koutra, Norun Hjertager Krog, Johanna Lepeule, Lea Maitre, Dries S. Martens, Oscar J. Pozo, Anjali Wijnhoven, Tim S. Nawrot, Martine Vrijheid, Oliver Robinson

**Affiliations:** a MRC Centre for Environment and Health, School of Public Health, Imperial College London, United Kingdom; b Department of Environmental Sciences, Vytautas Magnus University, Kaunas, Lithuania; c Bradford Institute for Health Research, Bradford Teaching Hospitals NHS Foundation Trust, Bradford, United Kingdom; d Department for Air Quality and Noise, Division for Climate and Environmental Health, Norwegian Institute of Public Health (NIPH), Oslo, Norway; e Applied Metabolomics Research Group, Hospital del Mar Research Institute, Barcelona, Spain; f ISGlobal, Barcelona, Spain; g Clinic of Preventive and Social Medicine, Medical School, University of Crete, Greece; h Department of Psychology, School of Social Sciences, University of Crete, Greece; i Université Grenoble Alpes, Inserm, CNRS, Team of Environmental Epidemiology Applied to Development and Respiratory Health, IAB, Grenoble, France; j Pompeu Fabra University, Barcelona, Spain; k CIBER de Epidemiología y Salud Pública (CIBERESP), Madrid, Spain; l Centre for Environmental Sciences, Hasselt University, Hasselt, Belgium; m Environmental & Health Unit, Leuven University, Belgium; n Mohn Centre for Children’s Health and Well-being, School of Public Health, Imperial College London, London, United Kingdom

**Keywords:** Socioeconomic position, Telomere length, Cortisol, Psychosocial stress, Family affluence

## Abstract

**Background:**

Shorter telomere length is associated with environmental stressors and has been proposed to underlie health inequalities in ageing trajectories. However, the relationship between socioeconomic position, psychosocial stress and telomere length is understudied in childhood, when ageing trajectories may be first defined. We aimed to examine the associations between family affluence, cortisol production and telomere length in a large cross-sectional study of European children.

**Methods:**

1160 children, aged 5–12 years, participating in the Human Early Life Exposome (HELIX) project, were recruited from cohorts in the UK, France, Spain, Norway, Lithuania, and Greece. Family material wealth was assessed using the international family affluence scale (FAS), psychosocial stress was defined by total urinary cortisol production, and leucocyte telomere length was measured through qPCR. Associations of FAS with cortisol production and telomere length were analysed using sequentially adjusted multivariable linear regression. The mediating role of cortisol production in the association between FAS and telomere length was examined using natural effects models.

**Findings:**

Compared to children of low FAS, children with high FAS had 4.94% (95% CI: 1.2%, 8.8%) longer telomeres after adjustment for sex, age, ethnicity and cohort. Estimates were similar upon further adjustment for perinatal, child health, and other socioeconomic factors. Additionally, children of medium and high FAS had significantly lower levels of cortisol production than children of low FAS (medium FAS: −20.8%, 95% CI: −31%, −8.5%; high FAS: −16.6% SD, 95% CI: −28%, −3.4%). However, cortisol production was not associated with telomere length, and no significant mediation of cortisol production and other tested mediators was found for the relationship between FAS and telomere length.

**Interpretation:**

The impacts of economic disadvantage are biologically observable in children and have implications for understanding health inequalities, both in child development and the onset of later age-related disease. Given the lack of mediation by cortisol production levels, as assessed via spot urine samples, further research should investigate alternative mechanisms underlying the association between affluence and telomere length.

**Funding:**

10.13039/100014013UK Research and Innovation (Grants: MR/S03532X/1, MR/Y02012X/1), European Community (Grants: 874583, 308333).

**Research in context**

**Evidence before this study**

Studies in adult populations suggest that low socioeconomic position and high psychosocial stress levels may be related to shorter telomeres. We searched PubMed on May 23, 2024, for any previously published literature on this relationship using the search terms “telomere, socioeconomic”, “telomere, stress”, “telomere, cortisol”, “socioeconomic, cortisol”, and “telomere, child∗” in order to identify studies in both child and adult populations on the subject. Notably, very few studies focused on children themselves, with most childhood studies looking retrospectively at telomere length in adults. Additionally, no identified studies examined a potential mediating effect of cortisol on the relationship between telomere length and socioeconomic position in children.

**Added value of this study**

This study sought to explore the association of family affluence with both telomere length, as a marker of cellular stress, and cortisol production, as an objective marker of acute psychosocial stress, in a large population of children. Our study is the largest of children examined for this purpose and included populations from six different European countries, increasing generalisability over previous studies. We show that increasing family affluence is associated with longer telomere lengths and reduced cortisol production. We further show that these effects are largely independent of family education level and social capital. Finally, we were able to conclude that in our sample, cortisol production was not a mediator in the relationship between family affluence and telomere length.

**Implications of all the available evidence**

The results of our study show that the impacts of socioeconomic position, as measured by family affluence are biologically observable in young children. This emphasises the importance of research into children themselves to better understand these impacts and appropriately develop interventions early in life. Overall, our study showed that a child’s early environment may have a significant effect on their future biological ageing trajectories.

## Introduction

Children born into families of disadvantaged socioeconomic position are at a greater risk of ill health and earlier mortality.^1^ The underlying mechanisms linking socioeconomic position in childhood and health remain unclear. Families of disadvantaged socioeconomic position have reduced access to financial and health resources, may be more exposed to physical or mental hazards, and are more likely to engage in unhealthy behaviours such as smoking.^1^ However, these factors appear to only partially explain observed health inequalities.^2^ Many of the health outcomes associated with disadvantaged socioeconomic position are also ageing-related and it has been proposed that a biological ageing mechanism could present a fundamental pathway underlying inequalities across multiple health outcomes.^3^

Telomere length is a widely used marker of biological age. The rate of shortening (attrition) varies with chronological age and is thought to be affected by both genetic and environmental factors.^4^ Individuals with shorter telomeres are at an increased risk for many adverse health outcomes, including shorter lifespan and increased risk of cancers, cardiovascular disease, and type 2 diabetes.^5^ Studies in adults have found that those of disadvantaged socioeconomic position may be at an increased risk of accelerated telomere attrition compared to their more affluent counterparts.^5^, ^6^, ^7^, ^8^ Notably, this relationship is understudied in children. Some studies have retroactively looked at the relationship between childhood socioeconomic position and telomere length in adults,^9^^,^^10^ but very few studies have examined children themselves, and those that have utilised relatively small sample sizes.^11^, ^12^, ^13^

Individuals of disadvantaged socioeconomic position may experience greater psychosocial stress due to increased exposure to chronic stressors, including unsafe neighbourhoods, job or housing instability, and food insecurity.^1^ Psychosocial stress has been associated with individual telomere length^14^ and increased rates of chronic disease, in particular cardiometabolic disorders.^15^ Following exposure to a stressor, the body may undergo allostasis, where physiological regulation is adjusted to the environment through the release of hormones and other signalling mechanisms.^16^ The hormone cortisol, which is released by the adrenal glands during allostasis^17^ can provide an objective measure of psychosocial stress.^18^ While the process of allostasis allows the body to adapt and respond to stress, sustained activation of this process may result in allostatic load or “biological wear and tear” on the body, which may cause increased cellular turnover and subsequent telomere shortening.^16^ In mouse models, induction of chronic stress led to chromosomal damage, as measured by histone phosphorylation^19^ that may promote the loss of telomere integrity, and to shorter telomeres.^20^ Several studies have reported associations between disadvantaged socioeconomic position and raised levels of cortisol, including in children, although results have been mixed,^18^^,^^21^ potentially due to differences in measurements of socioeconomic position and cortisol.^18^ Studies on the relationship between cortisol levels and telomere length are more limited.^17^ Bürgin et al. found young adults with a higher concentration of hair cortisol had significantly shorter telomeres,^22^ while de Punder et al. reported a negative association between telomere length and raised salivary cortisol in response to a laboratory stressor.^23^ Thus, it is possible that stress may mediate the relationship between individual socioeconomic position and telomere length.

In the present study, we aimed to analyse the relationship between family affluence and cortisol and leucocyte telomere length in a large pan-European study of children. We used the Family Affluence Scale (FAS) as our main indicator of socioeconomic position, since our cohort spanned six countries, and this measure was developed to more accurately capture socioeconomic position cross-culturally.^24^ We hypothesised that children of a lower family affluence would have shorter telomere length, and that this relationship would be mediated by greater cortisol production.

## Methods

### Study population

The study population included children participating in the European population-based HELIX exposome cohort, which was comprised of six ongoing longitudinal population-based birth cohorts from different European countries. These countries were the UK (Born in Bradford, BiB cohort), France (Étude des Déterminants pré et postnatals du développement et de la santé de l’Enfant, EDEN cohort), Spain (INfancia y Medio Ambiente, INMA cohort), Lithuania (Kaunas, KANC cohort), Norway (The Norwegian Mother, Father and Child Cohort Study, MoBa cohort), and Greece (RHEA Mother Child Cohort study, RHEA cohort).^25^

1301 subjects from the original cohorts took part in a harmonised ‘HELIX subcohort’ follow-up that occurred between December 2013 and February 2016. Children between the ages of 5 and 12 who had no serious health issues, and who had available data from the original study were selected for this sub-cohort with approximately equal representation from all six birth cohorts.^25^ Sample size for the HELIX subcohort was originally calculated to provide sufficient power to detect associations with a range of environmental exposures and child health outcomes. Questionnaire responses, blood and urine samples, and body composition measurements that were collected during a harmonised HELIX follow-up at each cohort centre, as well as relevant perinatal factors, were considered in this analysis.

### Ethics

Prior to the start of HELIX, all six cohorts had undergone the required evaluation by national ethics committees and obtained all the required permissions for their cohort recruitment and follow-up visits. Each cohort also confirmed that relevant informed consent and approval were in place for secondary use of data from pre-existing data. The work in HELIX was covered by new ethical approvals in each country and at enrolment in the new follow-up, participants were asked to sign a new informed consent form. Additionally, the current study was approved by the Imperial College Research Ethics Committee (Reference: 19IC5567).

### Family affluence scale

Our primary socioeconomic position indicator was the family affluence scale (FAS), designed for use in cross-country analyses to provide a universal and comparable measure of familial wealth.^24^ The FAS was derived from four questions answered by parents at the HELIX follow-up clinic, covering car ownership, sharing of children’s bedrooms, frequency of holidays and computer or tablet ownership, each scoring 0–2 points. Based on the sum of answers to these questions, FAS was categorised as low (score of 0, 1, 2), medium (score of 3, 4, 5), or high (score of 6+).^24^

### Cortisol and telomere measurements

At the HELIX follow up clinic, families were asked to bring two child spot urine samples (one last void before bedtime and one first morning void) collected in previously provided polypropylene tubes to the centre in cool packs and stored at −4 °C until processing. Children’s blood samples were collected into EDTA Vacutainer tubes at the end of the HELIX follow up clinic of the child to ensure an approximate 3 h fasting time since the last meal. Urine samples and buffy coat separated from the blood sample were then frozen at −80 °C under optimised and standardised procedures. Samples were then shipped from the HELIX centres to the analytical laboratories on dry ice.

Cortisol and related metabolites and creatinine were measured by liquid chromatography tandem mass spectrometry (LC-MS/MS) in urine samples^26^^,^^27^ at the Applied Metabolomics Research Laboratory, IMIM-Hospital del Mar Medical Research Institute, Spain. For operational reasons, cohort sample were run in separate batches. All analysis was conducted on the samples collected before bedtime on the night preceding the clinical visit to minimise the influence of diurnal variation. Briefly after addition of isotopic labelled internal standard, an enzymatic hydrolysis was performed to release the conjugated metabolites. Released steroids were then extracted by liquid–liquid extraction and determined by LC-MS/MS using a selected reaction monitoring method. Cortisol production (CP) was selected as our psychosocial stress measure since it assesses the total cortisol output. CP was calculated from the sum of the urinary sample’s cortisol value and ten glucocorticosteroid metabolites (20α-dihydrocortisol (20aDHF), 20β-dihydrocortisol (20bDHF), 5α,20α-cortol (5a20acortol), 5α,20β-cortol (5a20bcortol), 5α-tetrahydrocortisol (5aTHF), 5β,20α-cortol (5b20acortol), 5β,20β-cortol (5b20bcortol), 5β-dihydrocortisol (5bDHF), 5β-tetrahydrocortisol (5bTHF), 6β-hydroxycortisol (6OHF)). Individual metabolite coefficients of variation (CVs) ranged from 7 to 25% whereas the CV for CP was 16%.

DNA was extracted using the Chemagen kit (Perkin Elmer, USA, catalogue #: CMG-718) from the stored buffy coat. leucocyte average relative telomere length was measured by a modified qPCR protocol^28^ at a single laboratory at the Centre for Environmental Sciences, Hasselt University.^29^ All cohort samples were analysed together in a single analytical run. Telomere and single copy–gene reaction mixture, as well as PCR cycles used can be found in Martens et al.^29^ All measurements were performed in triplicate on a 7900HT Fast Real-Time PCR System (Applied Biosystems) in a 384-well format. On each run, a six-point serial dilution of pooled DNA and eight inter-run calibrators were also run to assess PCR efficiency and account for the inter-run variability. Relative telomere lengths were calculated using qBase Plus ver.3.4 software (Biogazelle, Zwijnaarde, Belgium) and were expressed as the ratio of telomere copy number to single-copy gene number (T/S) relative to the average T/S ratio of the entire sample set. We achieved CVs within triplicates of the telomere runs, single-copy gene runs, and T/S ratios of 0.84%, 0.43%, and 6.4%, respectively.

White blood cell proportions [CD4+ and CD8+ T cells, natural killer (NK) cells, monocytes, eosinophiles, neutrophils, and B cells] were estimated from DNA methylation data^30^ using the Reinius reference panel^31^ as implemented in meffil package.^32^

### Covariates

Demographic data included study cohort, sex (reported by parents), age, and ethnicity, which was grouped as “White”, “Pakistani or other Asian”, and “Other”, based on UK Census ethnicity categories and considering numbers within each category. Gestational age, recorded in weeks and calculated using the date of birth and the date of the mother’s last menstrual period, and child’s birth weight in kilograms, were extracted from medical records obtained from the local primary care centre, hospital delivery logs or child’s health cards. Smoking status of the mother during pregnancy was categorised as active, passive, or non-smoker.^25^

The child’s daily participation in moderate-to-vigorous physical activity (physical activities with intensity above three metabolic equivalent tasks) and sedentary activity was expressed in units of min/day. Physical activity and sedentary activity were measured using answers to a questionnaire developed by the HELIX research group.^25^ The KIDMED Mediterranean Diet Quality Index score was assigned based on 11 items from a food frequency questionnaire (ranging from −4 to 11, with higher scores reflecting greater adherence to a Mediterranean diet).^33^ Exposure to environmental tobacco smoke by the child was coded as a binary variable, and the Cohen’s shortened perceived stress score reported by the mother was calculated based on answers to questions regarding feeling in control, coping, and mental state.^34^ Fat mass was measured using bioelectric impedance analyses performed with the Bodystat 1500 (Bodystat Ltd.) equipment after 5 min of lying down. The proportion of fat mass was calculated using published age- and race-specific equations validated for use in children.^35^

Two further dimensions of socio-economic position were included as covariates: Family education level was highest based on the education levels of the child’s mother and father coded as primary school (1), secondary school (2), or university degree or higher (3), and these variables were summed to create a low (1–2), medium (3–4), or high (5–6) parental education score. Levels of family social capital were based on answers to questions regarding relationships with friends and family, participation in organisations or clubs, neighbourhood support and safety, and political party participation. This variable was coded as low, medium, or high based on tertiles of the scores.^25^

Missing values in the covariates (birth weight, gestational age, physical activity, KIDMED diet, fat mass, environmental tobacco smoke exposure, perceived stress, social capital and parental education) were treated as missing at random in the multivariable linear regression analyses and were thus imputed using the multivariate imputations by chained equations (MICE) package in R (version 4.2.1). MICE imputes missing data by assuming a distribution for each of our covariates and assigning plausible values for the missing data based on these distributions.^36^ We created a total of 5 imputed datasets, as is the default set by MICE, and used predictive mean matching as our imputation method. We set the max iterations to 25, and all other arguments in the model were set to default. Model results were subsequently pooled using the 5 imputed datasets.

### Statistics

**Fig. 1:**
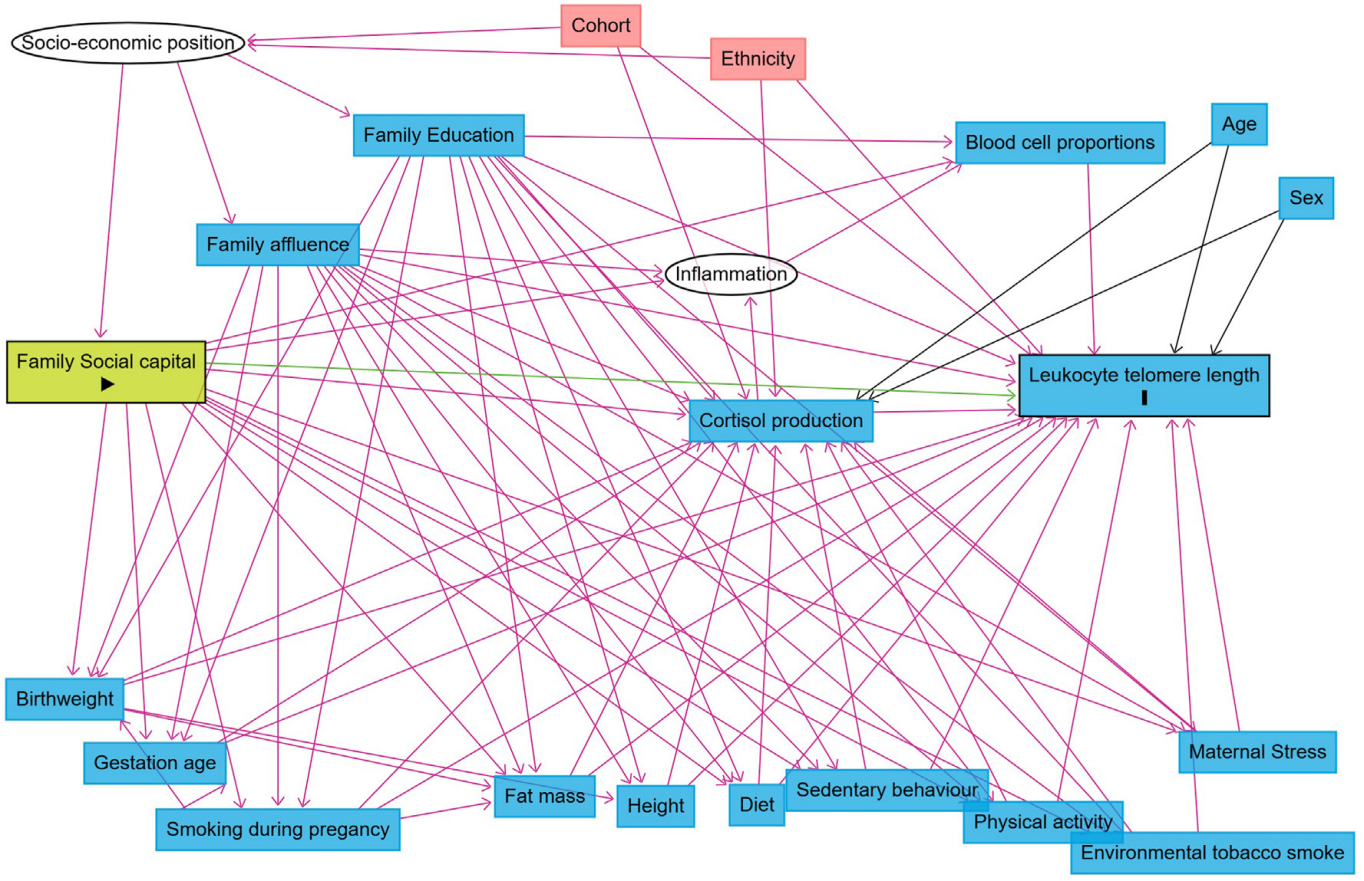
Directed acyclic graph (DAG) visualizing the causal assumptions in the relationship between family affluence and leucocyte telomere length. In this DAG, the exposure is family affluence (green box with a triangle) and the outcome is leucocyte telomere length (blue box with an “I”). White circles represent unobserved variables. Red arrows represent potential biasing paths, while green arrows represent potential causal paths for this DAG. The same causal assumptions were used when drawing DAGs for the models where cortisol production was the outcome (with family affluence) or exposure (with telomere length).

In cross-sectional analysis, the associations between FAS (independent variable) and both telomere length and cortisol production (dependent variables) were analysed with multivariable linear regressions. Cortisol production and telomere length were log-transformed. Covariates were preselected based on directed acyclic graphs (DAG, Fig. 1), using the DAGitty program,^37^ to visualise assumptions regarding biasing and causal pathways. We took a sequential approach to model adjustment to analyse the independent associations with FAS and explore the influence of different sets of covariates. Model 1 included only demographic factors (age, sex, ethnicity, and cohort) as the base model. Model 2 added two further dimensions of family socioeconomic position, social capital and parental education level. As we have assumed these three indicators represent interconvertible dimensions of socio-economic position, these adjustments represent the minimum adjustment set, selected based on the back door criterion, required to estimate the total association of FAS on both cortisol production and overall leucocyte telomere length. To estimate the direct association of FAS on cortisol production and overall leucocyte telomere length, we included two further models that additionally adjusted for covariates assumed to be on causal pathways. Model 3 adjusted for perinatal factors, including birth weight, gestational age, and prenatal smoke exposure while Model 4 further adjusted for postnatal child health and family environment factors, which included physical activity, sedentary behaviour, KIDMED diet score, fat mass, height, environmental tobacco smoke exposure, and maternal perceived stress. Perinatal and postnatal factors were added separately to explore their relative importance of these two timepoints on overall leucocyte telomere length. Non-linear relationships with age were tested through inclusion of an age^2^ term in our models. However, the term was not associated with the outcomes and did not alter estimates so was not subsequently included. Results are reported as percent differences between categories in the geometric mean in either cortisol production or telomere length, using the following formula (e^β^ −1) × 100%, where β is the coefficient from the regression model. We considered statistical significance to be a p-value of 0.05 or below.

In sensitivity analyses, we repeated analyses stratified by child sex and tested for interaction between sex and FAS using interaction terms. Since leukocytes represent a cell mixture, we further adjusted for cell proportions to estimate associations with “intrinsic” leucocyte telomere length, i.e., differences in telomere length within specific cell types. Associations with overall leucocyte telomere length may represent differences due to both intrinsic telomere length and cell composition. We fitted two models: the first model estimated the total association of family affluence with intrinsic leucocyte telomere length, through inclusion of all covariates in model 2 plus cell proportions (CD4+ and CD8+ T cells, natural killer (NK) cells, monocytes, eosinophiles, and B cells). The second model estimated the direct association of family affluence with intrinsic leucocyte telomere length, through inclusion of all covariates in model 4 plus cell proportions. Neutrophils were not included due to high multicollinearity with T cell proportions.

**Fig. 2:**
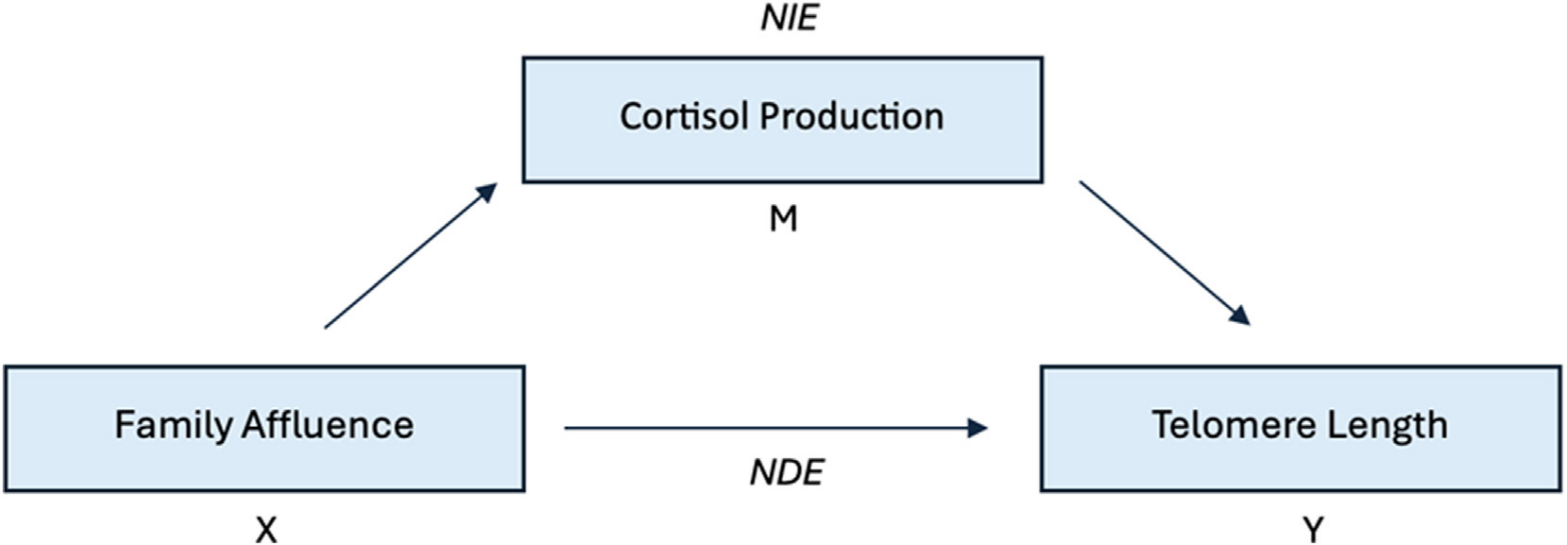
Natural direct effect (NDE) and natural indirect effect (NIE) of family affluence on telomere length, with cortisol production as the potential mediator.

To examine the potential mediating role of cortisol production in the relationship between FAS and telomere length, we first analysed the associations between cortisol production and telomere length. Model 1 included only demographic information as the base model (age, sex, ethnicity, and cohort). Model 2 presents the adjusted model for associations with overall leucocyte telomere length, and includes the minimum adjustment sets required for estimating both the direct and total associations (age, sex, ethnicity, and cohort, KIDMED score, environmental tobacco smoke, parental education, social capital, FAS, fat mass, maternal stress, physical activity and sedentary behaviour) according to the DAG (Fig. 1). Model 3 further adjusted for cell proportions to estimate direct and total associations with intrinsic leucocyte telomere length. To formally test and quantify any mediation, we further fitted natural effect models,^38^ using the imputation approach in the R package *medflex*.^39^ The purpose of mediation analysis is to quantify the natural direct effect (NDE), or effect of only X on Y (not mediated by a mediator (M)), and the natural indirect effect (NIE), or the effect of X on Y through M (Fig. 2). “Natural” effects represent the effect of X on Y when M is maintained at its “natural” level, observed when not exposed to the treatment and thus allowing for natural variation, and differs from “controlled” effects which describe outcomes at specific, fixed M values.^39^ All natural effect models were adjusted for confounders of both the exposure and mediator (selected based on the backdoor criterion) with overall leucocyte telomere length (age, sex, ethnicity, cohort, birthweight, gestational age, smoking during pregnancy, KIDMED score, environmental tobacco smoke, parental education, social capital, FAS, fat mass, height, maternal stress, physical activity and sedentary behaviour). To provide a comparison to the mediating role of cortisol production, we also examined the mediating roles of other postnatal covariates in separate mediation analyses. Additionally, natural killer, CD4+T, and CD8+T cells and cortisol production were included as covariates when testing for blood/urine-based biomarkers as potential mediators. Non-parametric bootstrapping with 1000 replications was used to calculate standard errors and 95% confidence intervals.

### Role of funders

The funding sources had no role in the study design, data collection, data analyses, interpretation, writing of the manuscript or the decision to submit it for publication.

## Results

### Study population

Of the 1301 children in the HELIX sub cohort, 1160 had available telomere length measurements and 975 children had cortisol production measurements. Sample characteristics of each subset were broadly similar (Table 1). Each cohort comprised 13–21% of the samples. There was a slight overrepresentation of males (55%) in both datasets. Mean age was eight years (range 5–12 years). 90% of children were of white ethnicity. In both samples, low FAS made up 9–12% of the data, while medium and high FAS made up 39–40% and 49–52% respectively. Similarly, this was a highly educated sample, with only 6–8% of parents falling into the “low” category for education, and 51–54% being considered “high education” in both samples.

**Table 1:** Demographics of the study population in telomere length and cortisol production subsets.

	Participants with telomere length data	Participants with cortisol production data
	N (%) or Median [IQR]	N (%) or Median [IQR]
**N**	1160	975
**Telomere length (T/S ratio)**^a^	0.996 [0.881, 1.12]	0.992 [0.884, 1.12]
Missing	–	18 (1.8%)
**Cortisol production (μg/μmol creatinine)**	0.279 [0.167, 0.529]	0.281 [0.167, 0.529]
Missing	203 (17.5%)	–
**Demographic factors**		
Cohort		
BIB	200 (17.2%)	132 (13.5%)
EDEN	145 (12.5%)	134 (13.7%)
INMA	211 (18.2%)	203 (20.8%)
KANC	195 (16.8%)	178 (18.3%)
MOBA	211 (18.2%)	200 (20.5%)
RHEA	198 (17.1%)	128 (13.1%)
Sex		
Male	638 (55.0%)	540 (55.4%)
Female	522 (45.0%)	435 (44.6%)
Age	7.25 [6.49, 8.85]	8.11 [6.51, 8.93]
Ethnicity		
White	1037 (89.4%)	881 (90.4%)
Pakistani or other Asian	96 (8.3%)	65 (6.7%)
Other	27 (2.3%)	21 (2.2%)
**Perinatal factors**		
Birth weight (kg)	3.38 [3.05, 3.7]	3.39 [3.08, 3.72]
Missing	10 (0.9%)	9 (0.9%)
Gestational age (weeks)	40 [38.7, 40.7]	40 [38.9, 40.7]
Missing	10 (0.9%)	9 (0.9%)
Prenatal smoke exposure		
None	615 (53.0%)	541 (55.5%)
Passive only	371 (32.0%)	294 (30.2%)
Active	174 (15.0%)	140 (14.4%)
**Child factors**		
Moderate to vigorous physical activity (min/day)	41.4 [0, 296]	42.9 [17.1, 75]
Missing	29 (2.5%)	22 (2.3%)
Sedentary behaviour (min/day)	214 [156, 289]	212 [154, 289]
Missing	8 (0.7%)	7 (0.7%)
KIDMED diet score	3 [2, 4]	3 [2, 4]
Missing	3 (0.3%)	3 (0.3%)
Fat mass percentage (%)	22.4 [17.5, 27.8]	22.4 [17.3, 27.8]
Missing	9 (0.8%)	7 (0.7%)
Height (m)	1.27 [1.2, 1.35]	1.29 [1.21, 1.36]
Environmental tobacco smoke exposure		
No	722 (62.2%)	628 (64.4%)
Yes	411 (35.4%)	324 (33.2%)
Missing	27 (2.3%)	23 (2.4%)
Perceived stress score	4 [2, 7]	4 [2, 6]
Missing	4 (0.3%)	3 (0.3%)
**Socioeconomic position**		
Family affluence score		
Low	133 (11.5%)	91 (9.3%)
Medium	462 (39.8%)	382 (39.2%)
High	565 (48.7%)	502 (51.5%)
Family social capital		
Low	513 (44.2%)	418 (42.9%)
Medium	264 (22.8%)	228 (23.4%)
High	298 (25.7%)	256 (26.3%)
Missing	85 (7.3%)	73 (7.5%)
Parental education		
Low	87 (7.5%)	61 (6.3%)
Middle	388 (33.4%)	324 (33.2%)
High	588 (50.7%)	523 (53.6%)
Missing	97 (8.4%)	67 (6.9%)

a Telomere lengths are expressed as ratios of telomere copy number to single-copy gene number.

### Association between family affluence and telomere length

Four models analysing the association between FAS and telomere length, with sequential addition of covariates, are presented in Table 2. In the basic Model 1, children with a high FAS had 4.94% longer telomeres [95% Confidence interval (CI): 1.2%, 8.8%] than children with a low FAS. Children with a medium FAS had 2.55% [95% CI: −(−1%, 6.2%)] longer telomeres than children with a low FAS although this was not statistically significant. Similar associations were observed in all four models. In Model 2, we tested whether associations with FAS were independent of other dimensions of socioeconomic position through additional adjustment for parental education level and family social capital, observing a similar estimate of 4.78% longer telomeres [95% CI: 0.7%, 9%] in high FAS children compared to FAS. Minimal attenuation was observed when further adjusted for perinatal factors in Model 3. In Model 4, which adjusted for further child-related factors and represents the direct association of FAS with overall leucocyte telomere length, we again found the association to be only slightly attenuated, with high FAS children having 4.7% [95% CI: 0.61%, 9%] longer telomeres than low FAS children. We also observed a trend for 2.24% [95% CI: −0.6%, 5.2% in Model 2] longer telomeres in children of families with high social capital. There was little evidence for an association between parental education and telomere length.

**Table 2:** Sequentially adjusted models examining the relationship between family affluence scale and telomere length (n = 1,160).

Telomere length	Model 1 (Basic model)		Model 2 (+socioeconomic position factors)		Model 3 (+socioeconomic position and perinatal factors)		Model 4 (+socioeconomic position, perinatal and child factors)	
	% changes (95% CI)	p-value	% changes (95% CI)	p-value	% changes (95% CI)	p-value	% changes (95% CI)	p-value
Family affluence scale–Low	–	–	–	–	–	–	–	–
Family affluence scale–Medium	2.55% (−1.0%, 6.2%)	0.16	2.52% (−1.3%, 6.4%)	0.20	2.45% (−1.3%, 6.4%)	0.21	2.35% (−1.4%, 6.3%)	0.23
Family affluence scale–High	4.94% (1.2%, 8.8%)	0.0098	4.78% (0.7%, 9%)	0.021	4.64% (0.56%, 8.9%)	0.025	4.70% (0.61%, 9.0%)	0.024
Sex–Male	–	–	–	–	–	–	–	–
Sex–Female	5.09% (2.9%, 7.3%)	<0.0001	4.99% (2.8%, 7.2%)	<0.0001	5.19% (3.0%, 7.4%)	<0.0001	5.61% (3.3%, 8.0%)	<0.0001
Age (years)	−2.26% (−4.5%, 0.011%)	0.051	−2.32% (−4.6%, −0.028%)	0.047	−2.31% (−4.6%, −0.015%)	0.049	−1.73% (−4.2%, 0.84%)	0.18
Cohort–BIB	–	–	–	–	–	–	–	–
Cohort–EDEN	9.78% (−1.3%, 22%)	0.085	10.1% (−1.2%, 23%)	0.081	10.4% (−0.89%, 23%)	0.072	9.45% (−1.9%, 22%)	0.11
Cohort–INMA	4.98% (−1.8%, 12%)	0.15	5.55% (−1.3%, 13%)	0.12	6.15% (−0.82%, 14%)	0.085	5.93% (−1.1%, 13%)	0.099
Cohort–KANC	1.51% (−2.9%, 6.1%)	0.51	1.98% (−2.7%, 6.8%)	0.41	2.13% (−2.6%, 7.1%)	0.38	2.73% (−2.4%, 8.1%)	0.30
Cohort–MOBA	7.07% (0.76%, 14%)	0.028	6.63% (0.033%, 14%)	0.049	6.42% (−0.18%, 13%)	0.057	5.25% (−1.4%, 12%)	0.13
Cohort–RHEA	6.65% (2.0%, 11%)	0.0043	6.82% (2.0%, 12%)	0.0047	8.14% (2.9%, 14%)	0.0020	7.93% (2.5%, 14%)	0.0037
Ethnicity–White	–	–	–	–	–	–	–	–
Ethnicity—Pakistani or other Asian	5.41% (0.29%, 11%)	0.038	5.49% (0.34%, 11%)	0.036	5.95% (0.66%, 12%)	0.027	6.77% (1.4%, 12%)	0.014
Ethnicity–Other	6.61% (−1.1%, 15%)	0.095	6.70% (−1.1%, 15%)	0.092	6.99% (−0.81%, 15%)	0.080	7.30% (−0.54%, 16%)	0.069
Parental education–Low			–	–	–	–	–	–
Parental education–Middle			−0.712% (−5.0%, 3.8%)	0.75	−0.843% (−5.1%, 3.7%)	0.71	−0.558% (−4.9%, 3.9%)	0.80
Parental education–High			−0.545% (−5.1%, 4.2%)	0.82	−0.878% (−5.4%, 3.9%)	0.71	−0.791% (−5.4%, 4.0%)	0.74
Family social capital–Low			–	–	–	–	–	–
Family social capital–Medium			0.959% (−1.8%, 3.8%)	0.50	0.884% (−1.9%, 3.7%)	0.53	0.809% (−2.0%, 3.7%)	0.57
Family social capital–High			2.24% (−0.60%, 5.2%)	0.12	2.13% (−0.72%, 5.1%)	0.14	2.27% (−0.63%, 5.3%)	0.13
Birth weight (kg)					1.59% (−1.0%, 4.3%)	0.23	1.94% (−0.76%, 4.7%)	0.16
Gestational age (weeks)					0.0739% (−0.70%, 0.85%)	0.85	0.00502% (−0.78%, 0.79%)	0.99
Prenatal smoke exposure–None					–	–	–	–
Prenatal smoke exposure -Passive					−1.18% (−3.9%, 1.6%)	0.40	−1.02% (−3.8%, 1.8%)	0.48
Prenatal smoke exposure–Active					−0.983% (−4.3%, 2.4%)	0.57	−0.563% (−4.0%, 3.0%)	0.75
Moderate-to-vigorous physical activity (min/day)							−0.0231% (−0.051%, 0.0046%)	0.10
Sedentary behaviour (min/day)							0.00338% (−0.0056%, 0.012%)	0.46
KIDMED diet score							0.412% (−0.24%, 1.1%)	0.21
Fat mass percentage of total weight (%)							−0.177% (−0.33%, −0.023%)	0.025
Height (m)							−6.24% (−24%, 15%)	0.54
Environmental tobacco smoke exposure–No							–	–
Environmental tobacco smoke exposure–Yes							−0.219% (−2.6%, 2.3%)	0.86
Maternal perceived stress score							0.0608% (−0.33%, 0.45%)	0.76

Each model is adjusted for all covariates shown in respective columns.

Additionally, females were found to have significantly longer telomere length than males across all models [5.56%, 95% CI: 3.3%, 8% in Model 4]. Children from the Greek RHEA cohort were also found to have significantly longer telomeres, compared to children in the BiB cohort, across all models. Children with additional fat mass had shorter telomeres by 0.18% [95% CI: −0.33%, −0.023%, Model 4] for each percentage increase in fat mass.

### Adjustment for cell proportions

Adjustment for cell proportions led to some attenuation of the association between family affluence and telomere length. In a model adjusted for demographic factors, parental education level, social capital, perinatal and child-related factors (i.e., Model 4) plus cell proportions, we observed 4.06% [95% CI: 0.0067%, 8.3%] longer telomeres in high FAS compared to low FAS children (Supplementary Table S1).

### Association between family affluence and cortisol production

We used the same adjustment sets (Models 1–4) to test the association between FAS and cortisol production values in 975 children (Table 3). In all models, children in the middle FAS category had significantly reduced cortisol production compared to children with a low FAS. In the basic Model 1, children from medium FAS families had 20.8% lower cortisol production [95% CI: −31%, −8.5%] than children from low FAS families. Children with a high FAS score also had lower cortisol production than children with a low FAS score [Model 1: −16.6%, 95% CI: −28%, −3.4%]. Interestingly, estimates were slightly increased upon further adjustment for other socioeconomic position factors, with medium FAS and high FAS children estimated to have 22.8% lower cortisol production [95% CI: −33%, −10%] and 18.8% lower cortisol production [95% CI: −30%, −5.1%] respectively in Model 2. This difference between models was likely driven by the inclusion of parental education, with a trend observed for 15.2% [95% CI: −3.4%, 37%] higher cortisol production levels among children of high parental education levels compared to low parental education levels. Little change was observed upon adjustment for perinatal factors in Model 3 and further adjustment for child factors in Model 4.

**Table 3:** Sequentially adjusted models examining the relationship between family affluence scale and cortisol production (n = 975).

Cortisol production	Model 1 (Basic model)		Model 2 (+socioeconomic position factors)		Model 3 (+socioeconomic position and perinatal factors)		Model 4 (+socioeconomic position, perinatal and child factors)	
	% changes (95% CI)	p-value	% changes (95% CI)	p-value	% changes (95% CI)	p-value	% changes (95% CI)	p-value
Family affluence scale–Low	–	–	–	–	–	–	–	–
Family affluence scale–Medium	−20.8% (−31%, −8.5%)	0.0015	−22.8% (−33%, −10%)	0.00071	−22.5% (−33%, −10%)	0.00086	−22.5% (−33%, −10%)	0.00084
Family affluence scale–High	−16.6% (−28%, −3.4%)	0.016	−18.8% (−30%, −5.1%)	0.0088	−18.8% (−31%, −5.0%)	0.0092	−17.9% (−30%, −4.1%)	0.013
Sex–Male	–	–	–	–	–	–	–	–
Sex–Female	3.15% (−4.6%, 12%)	0.44	2.93% (−4.8%, 11%)	0.47	3.83% (−4.2%, 12%)	0.36	0.906% (−7.2%, 9.7%)	0.83
Age (years)	−4.03% (−12%, 4.4%)	0.34	−3.70% (−12%, 4.8%)	0.38	−3.65% (−12%, 4.9%)	0.39	−1.53% (−10%, 8.1%)	0.75
Cohort–BIB	–	–	–	–	–	–	–	–
Cohort–EDEN	−66.2% (−77%, −50%)	<0.0001	−67.8% (−78%, −52%)	<0.0001	−67.3% (−78%, −51%)	<0.0001	−66.2% (−78%, −49%)	<0.0001
Cohort–INMA	−68.4% (−76%, −59%)	<0.0001	−69.3% (−76%, −60%)	<0.0001	−68.8% (−76%, −59%)	<0.0001	−67.7% (−75%, −58%)	<0.0001
Cohort–KANC	−54.1% (−62%, −45%)	<0.0001	−55.7% (−63%, −47%)	<0.0001	−55.7% (−63%, −46%)	<0.0001	−59.2% (−67%, −50%)	<0.0001
Cohort–MOBA	−64.4% (−72%, −55%)	<0.0001	−65.7% (−73%, −56%)	<0.0001	−65.8% (−73%, −56%)	<0.0001	−62.5% (−71%, −52%)	<0.0001
Cohort–RHEA	−4.00% (−20%, 16%)	0.67	−7.44% (−24%, 12%)	0.43	−5.24% (−23%, 17%)	0.62	−4.41% (−23%, 19%)	0.68
Ethnicity–White	–	–	–	–	–	–	–	–
Ethnicity—Pakistani or other Asian	14.9% (−6.6%, 41%)	0.19	15.2% (−6.5%, 42%)	0.18	17.1% (−5.4%, 45%)	0.15	13.0% (−9.0%, 40%)	0.27
Ethnicity–Other	−6.19% (−30%, 26%)	0.67	−4.77% (−29%, 28%)	0.75	−3.94% (−29%, 30%)	0.79	−7.62% (−32%, 25%)	0.60
Parental education–Low			–	–	–	–	–	–
Parental education–Middle			16.1% (−2.2%, 38%)	0.088	15.6% (−2.7%, 37%)	0.098	15.5% (−3.0%, 37%)	0.11
Parental education–High			15.2% (−3.4%, 37%)	0.11	14.2% (−4.4%, 36%)	0.14	18.1% (−1.5%, 42%)	0.072
Family social capital–Low			–	–	–	–	–	–
Family social capital–Medium			3.07% (−6.9%, 14%)	0.56	3.18% (−6.9%, 14%)	0.55	4.92% (−5.4%, 16%)	0.36
Family social capital–High			−1.63% (−11%, 9.2%)	0.76	−1.56% (−11%, 9.4%)	0.77	0.766% (−9.7%, 12%)	0.89
Birth weight (kg)					5.18% (−4.7%, 16%)	0.32	5.33% (−4.9%, 17%)	0.32
Gestational age (weeks)					0.247% (−2.7%, 3.3%)	0.87	0.261% (−2.7%, 3.3%)	0.86
Prenatal smoke exposure–None					–	–	–	–
Prenatal smoke exposure–Passive					−0.0918% (−10%, 11%)	0.99	−1.48% (−11%, 9.6%)	0.78
Prenatal smoke exposure–Active					−3.57% (−15%, 9.8%)	0.58	−7.46% (−19%, 5.8%)	0.26
Moderate-to-vigorous physical activity (min/day)							0.0486% (−0.055%, 0.15%)	0.36
Sedentary behaviour							0.0291% (−0.0049%, 0.063%)	0.094
KIDMED diet score							−2.82% (−5.1%, −0.44%)	0.021
Fat mass percentage of total weight (%)							0.825% (0.24%, 1.4%)	0.0058
Height (m)							−45.6% (−74%, 16%)	0.12
Environmental tobacco smoke exposure–No							–	–
Environmental tobacco smoke exposure–Yes							−0.891% (−9.7%, 8.8%)	0.85
Maternal perceived stress score							0.873% (−0.64%, 2.4%)	0.26

Each model is adjusted for all covariates shown in respective columns.

Significant differences were observed in cortisol production levels across cohort, with higher levels observed in the RHEA children and BiB cohorts. Other demographic and prenatal variables were not found to be significantly associated with cortisol production. A greater KIDMED diet score was also associated with approximately 2.82% decreased cortisol production [95% CI: −5.1%, −0.44% in Model 4]. Fat mass was associated with approximately 0.83% increased cortisol production [95% CI: 0.24%, 1.4% in Model 4].

### Sex-stratified analysis

Associations of FAS with telomere length and with cortisol production were similar in boys and girls (Table 4). Interaction terms for sex and FAS were non-significant for telomere length (Model 4, p value for interaction with high FAS = 0.35) and cortisol production (Model 4, p value for interaction with high FAS = 0.26).

**Table 4:** Sequentially adjusted models examining the relationships between family affluence scale (FAS) and telomere length and cortisol production, stratified by child sex.

	Model 1 (Basic model)		Model 2 (+socioeconomic position factors)		Model 3 (+socioeconomic position and perinatal factors)		Model 4 (+socioeconomic position, perinatal and child factors)	
	% change (95% CI)	p-value	% change (95% CI)	p-value	% change (95% CI)	p-value	% change (95% CI)	p-value
**Telomere length**								
Girls (n = 522)								
FAS—Low	–	–	–	–	–	–	–	–
FAS—Medium	3.25% (−1.8%, 8.6%)	0.21	4.06% (−1.4%, 9.8%)	0.15	3.82% (−1.7%, 9.6%)	0.17	3.58% (−1.9%, 9.4%)	0.21
FAS—High	4.40% (−0.90%, 10%)	0.10	4.96% (−0.85%, 11%)	0.095	4.75% (−1.1%, 11%)	0.11	4.84% (−1.0%, 11%)	0.11
Boys (n = 638)								
FAS—Low	–	–	–	–	–	–	–	–
FAS—Medium	2.25% (−2.7%, 7.4%)	0.38	1.51% (−3.7%, 7.0%)	0.58	1.52% (−3.7%, 7.0%)	0.57	2.01% (−3.2%, 7.5%)	0.46
FAS—High	5.56% (0.26%, 11%)	0.039	4.63% (−1.0%, 11%)	0.11	4.46% (−1.2%, 10%)	0.12	5.23% (−0.47%, 11%)	0.073
**Cortisol production**								
Girls (n = 435)								
FAS—Low	–	–	–	–	–	–	–	–
FAS—Medium	−15.3% (−32%, 5.4%)	0.14	−19.2% (−36%, 1.7%)	0.069	−18.5% (−35%, 2.7%)	0.083	−17.5% (−35%, 4.3%)	0.11
FAS—High	−12.0% (−30%, 10%)	0.26	−16.3% (−34%, 6.1%)	0.14	−15.6% (−34%, 7.3%)	0.17	−14.1% (−33%, 9.7%)	0.22
Boys (n = 540)								
FAS—Low	–	–	–	–	–	–	–	–
FAS—Medium	−24.8% (−38%, −8.7%)	0.0041	−25.7% (−39%, −9.2%)	0.0038	−25.7% (−39%, −9.0%)	0.0041	−26.9% (−40%, −11%)	0.0024
FAS—High	−20.3% (−35%, −2.7%)	0.026	−21.7% (−37%, −3.4%)	0.022	−22.1% (−37%, −3.7%)	0.021	−22.4% (−37%, −4.3%)	0.018

Model 1 adjusted for age, sex, ethnicity, and cohort. Model 2 adjusted for age, sex, ethnicity, cohort, social capital and parental education level. Model 3 adjusted for age, sex, ethnicity, cohort, social capital, parental education level, birth weight, gestational age, and prenatal smoke exposure. Model 4 adjusted for age, sex, ethnicity, cohort, social capital, parental education level, birth weight, gestational age, prenatal smoke exposure, physical activity, sedentary behaviour, KIDMED diet score, fat mass, height, environmental tobacco smoke exposure, and maternal perceived stress.

### Mediation analysis

We did not observe a significant direct association between cortisol production and telomere length (−0.4% %, 95% CI: −3.1%, 2.4%, adjusted for age, sex, cohort and ethnicity, Supplementary Table S2). Furthermore, in formal mediation analysis (Supplementary Fig. S2), the NIE (i.e., the portion of the total effect of FAS on telomere length, mediated by the mediating factor) through cortisol production was found to be null [NIE = 0.00%; 95% CI: −0.18%, 0.19%]. As a comparison, we additionally tested mediation effects of other child health factors and cell proportions. All mediating effects were found to be non-significant and close to zero. The largest NIE, albeit still non-significant, was observed for the proportion of natural killer cells, with an estimated NIE of 0.46% [95% CI: −0.36%, 1.30%].

## Discussion

In a large sample of European children, we examined whether family affluence is associated with telomere length, a marker of cellular stress and key biological ageing mechanism, and cortisol production, a marker of the physiological response to acute external stress. We found that children from families of high family affluence had approximately 5% longer leucocyte telomeres than children from families of low family affluence. Although the telomere length assay we used was based on a relative method, we have estimated this difference in terms of telomeric year to illustrate the public health significance of this difference. Based on reported an average telomere length in children of 8 kb and annual telomere loss of 40 bp,^40^ we estimate that children of low family affluence are on average approximately 10 years older biologically than their high family affluence peers, based on extrapolation to telomeric year equivalence. We further found that children of both medium and high family affluence had lower cortisol production than children of low family affluence. However, we did not observe a dose response across affluence categories, suggesting that low affluence (i.e., scarcity) is the main driver of differences in acute external stress as indicated by cortisol production. We sequentially adjusted our analyses for a range of factors including additional dimensions of socioeconomic position, that may act as confounders or potential mediators, and observed broadly similar estimates across models, indicating that associations with family affluence were largely independent of these covariates. In mediation analysis, we did not observe any evidence for a mediating effect by urinary cortisol production on the relationship between family affluence and telomere length.

In adults, associations between socioeconomic position and telomere length have been mixed, with stronger evidence for an association between higher education, a measure of socioeconomic position in early adult life, and longer telomeres.^41^ Previous studies regarding the effects of socioeconomic position in children are limited, of generally small sample size and have used a variety of measures of socioeconomic position. All of the five studies we identified in children or newborns reported a positive relationship between socioeconomic advantage and telomere length. In Kenya, Needham et al. found that children from families with more livestock holdings, a measure of wealth, had longer telomeres than those from families with less livestock holdings.^11^ In a sample of 70 U.S. children aged 7–13, Needham et al. found that children whose parents had never attended college had shorter telomere length equivalent to an additional six years of ageing on average compared to children with at least one college-educated parent.^12^ Similarly, a study of 40 African American boys showed that those with low family income, unstable family structures, and low maternal education levels had significantly shorter telomeres compared to those living in more advantageous environments.^13^ In a study of 92 children in Hong Kong found that children whose families reported greater financial strain had significantly shorter telomeres, and that this difference was more pronounced in children from high-income families.^42^ One study in Belgium, found that a composite measure of socioeconomic position was positively associated with telomere length in newborn boys, but not girls.^43^ We did not observe an interaction between sex and FAS on the association with telomere length in our study. These studies reflect the importance of capturing socioeconomic position appropriately within the cultural context of the study. Our primary indicator of socioeconomic position was the FAS, which was first created by the WHO Health Behaviour in School-Aged Children Study of 35 countries to address the varying definitions of socioeconomic position across countries and cultures.^24^

FAS relates to the economic capital of the family, and we assessed it independently of other dimensions of socioeconomic position, including family social capital and parental education, which relates to family cultural capital.^44^ When adjusted for these dimensions, the relationship between FAS and telomere length was slightly attenuated, which appeared to be driven mainly by high family social capital in the mutually adjusted model. Social capital, the collection of material and immaterial resources associated with belonging to a group, has not been studied before in this context. Interestingly, the association between FAS and lower cortisol production was in fact slightly strengthened upon inclusion of other socioeconomic position dimensions and appeared driven in this instance by high parental education in the mutually adjusted model. However, the associations between social capital and parental education with telomere length and cortisol production respectively did not reach statistical significance in the mutually adjusted models.

The association between lower FAS and greater cortisol production we observe may reflect the initial physiological response to greater early-life adversity experienced by children of disadvantaged socioeconomic position.^45^ A recent review identified four studies that reported an association between lower family income and higher hair cortisol levels in children, although nine other, mainly smaller, studies included in the review reported no association.^21^ Furthermore, a systematic review of 11 studies in children found that all studies reported a negative relationship between early adversity and telomere length.^46^ We therefore asked if cortisol production may present a mechanistic pathway linking family affluence to differences in telomere length. Our analysis found that the association between FAS and telomere length was not mediated by cortisol production. This may be due to telomere length providing a cumulative indicator of cellular stress over the life course, while urinary cortisol production measures may better reflect acute or recent psychosocial stress than chronic stress.^47^ We therefore cannot exclude the possibility that chronically raised levels of cortisol production may mediate the relationship between family affluence and telomere length. Similarly, we found the association of FAS and telomere length to be largely independent of health behaviours such as diet and physical activity. While several studies in adults have found that the impacts of psychosocial stress and socioeconomic position on telomere length are mediated by positive health behaviours such as a balanced diet and increased physical activity,^48^^,^^49^ we found smaller effects of these covariates in our analysis of children. Although we endeavoured to include a broad range of well-measured covariates in our analysis, we were unable to demonstrate important pathways linking FAS to telomere length. It is likely that multiple unmeasured factors, such as pollution, specific nutrients or inflammation for example, contribute to this relationship. Indeed, we observed the largest attenuation in our estimates upon adjustment for leukocytes cell proportions, indicating the potential importance of inflammation in the relationship between family affluence and leucocyte telomere length, which should be explored further. Furthermore, it has been shown that newborn telomere length is strongly predictive of telomere length measured in children at 4 years of age, suggesting that telomere length in children may already be largely set at birth.^50^ In fact, we observed a larger, albeit still modest, attenuation in the association between FAS and telomere length upon adjustment for perinatal factors than for subsequent adjustments for postnatal factors, indicating the important role of the prenatal environment for childhood telomere length.

We observed significant differences in measured cortisol production levels and telomere length between cohorts. While these differences likely reflect meaningful biological variation between the cohorts resulting for instance from differing environments in each country, they may also partially result from analytical and pre-analytical technical variation. Pre-analytical variation was controlled as best as possible using harmonised sample collection and processing protocols within each centre. Analytical variation was controlled through use of single laboratories, running of cohorts in a single run for the telomere assay, and use of quantified assays for the cortisol and steroid determination. However, cohorts were analysed separately for the measurement of cortisol and related metabolites which may have introduced some analytical variation. Nevertheless, this was controlled through adjustment for cohort as a fixed effect in all analyses.

Our study had some other limitations. The HELIX cohort was overall more affluent and well educated than the general population, which may have decreased the statistical power of our analysis and limited generalisability. The study was primarily a cross-sectional design, although reverse causality was unlikely between FAS and our outcomes. We cannot exclude unmeasured confounding as well as measurement error for certain confounders, such as prenatal smoke exposure. Furthermore, our measure of cortisol production was made in urine, allowing us only to make inferences regarding acute stress at the time of the study and, while this may reflect habitually raised cortisol production levels, we were unable to examine earlier or prolonged stress or adversity, which may be more relevant for effects on telomere length. Future research using repeat measurements of hair cortisol, which assesses levels over approximately three months, may be better suited to address the role of prolonged stress on telomere length.

Our study also had several important strengths. We analysed the association of both socioeconomic position and psychosocial stress levels, in the form of cortisol, with telomere length in a large cohort of children. The sample size and inclusion of children from six European countries increases the generalisability of the results. We included extensive data on several factors relating to demographics, lifestyle, familial capital, and biological samples so we could conduct a thorough and comprehensive analysis and were also able to consider several other mediators in the relationship besides cortisol production. Furthermore, our measure of cortisol production included multiple cortisol metabolites which improves the clinical accuracy of cortisol assessment, compared to cortisol alone,^47^ and urine samples were taken at a unified time as the last void before bedtime to minimise effects of diurnal variation. The use of urine as a sample matrix has the advantage of ease of sampling, including at home at specified times, and is less prone to contamination than salivary samples and often more acceptable to participants than hair sampling. Finally, we used a universal measure of socioeconomic position to minimise variation due to cross-cultural differences.

In conclusion, we have demonstrated in a large, pan-European cohort that children of low affluence families have shorter telomeres and greater recent cortisol production than their more advantaged peers. These findings indicate that detrimental effects of lower familial affluence are biologically observable in children. Since telomere length is a primary biological ageing mechanism and prolonged psychosocial stress may induce greater “wear and tear” on multiple biological systems through allostasis, our results have important implications for understanding health inequalities, both in childhood and for the onset of later age-related disease. Public health policy should focus on interventions, such as the Sure Start program for example,^51^ that mitigate adverse environments for all children, early in life, to reduce the burden of mortality and age-related disease.

## Contributors

OR conceived and acquired funding for the study, contributed to project supervision and writing of the original draft. KM performed data analysis and contributed to the writing of the original draft. CEL contributed to the formal data analysis, accessed and verified the underlying data, and contributed to project supervision. AW assisted with data analysis and figure preparation. OP and NH contributed to the analysis the cortisol measurements. TN and DM performed the telomere length analysis and assisted with its interpretation. LM and MV contributed to the resource management and data curation at the HELIX consortium. SA, SB, RG, KBG, MK, KK, NHK, JL contributed to the resource management or data curation in the participating study cohorts. All authors contributed to the editing and reviewing of the manuscript, and all have read and approved the final manuscript version.

## Data sharing statement

Due to data protection regulations in each participating country and participant data use agreements, human subject data used in this project cannot be freely shared. The raw data supporting the current study are available on request subject to ethical and legislative review. The “HELIX Data External Data Request Procedures” are available with the data inventory in this website: https://athleteproject.eu/helix-cohort/. The document describes who can apply to the data and how, the timings for approval and the conditions to data access and publication. Researchers who have an interest in using data from this project for reproducibility or in using data held in general in the HELIX data warehouse for research purposes can apply for access to data. Interested researchers should fill in the application protocol found in ANNEX I at https://www.projecthelix.eu/files/helix_external_data_request_procedures_final.pdf and send this protocol to helixdata@isglobal.org. The applications are received by the HELIX Coordinator, and are processed and approved by the HELIX Project Executive Committee.

## Declaration of interests

All authors declare that they have no conflicts of interest.
