# Supplementary material for: Associations of family affluence with cortisol production and telomere length in European children
Source: eBioMedicine. 2025 Jun 5;117:105793. doi: 10.1016/j.ebiom.2025.105793 (PMC12278409; doi:10.1016/j.ebiom.2025.105793)
Supplement: Supplementary Fig. S2 and Tables S1 and S2 [file mmc1.docx]

**Table S1 Effect of family affluence scale on telomere length adjusting for DNA methylation estimated immune cell proportions. Each model is adjusted for all covariates shown in respective columns.**

| **Telomere length** | **Model S1 Total effect of FAS on intrinsic leucocyte telomere length** | | **Model S2 Direct effect of FAS on intrinsic leucocyte telomere length** | |
| --- | --- | --- | --- | --- |
|  | **% changes (95% CI)** | **P-value** | **% changes (95% CI)** | **P-value** |
| **Family Affluence Scale - Low** | - | - | - | - |
| **Family Affluence Scale - Medium** | 2.26% (-1.5%, 6.2%) | 0.24 | 2.14% (-1.6%, 6%) | 0.27 |
| **Family Affluence Scale - High** | 4.1% (0.064%, 8.3%) | 0.046 | 4.06% (0.0067%, 8.3%) | 0.050 |
| **Sex - Male** | - | - | - | - |
| **Sex - Female** | 5.33% (3.1%, 7.6%) | <0.0001 | 5.79% (3.5%, 8.2%) | <0.0001 |
| **Age (years)** | -2.4% (-4.6%, -0.13%) | 0.039 | -1.95% (-4.4%, 0.61%) | 0.13 |
| **Cohort - BIB** | - | - | - | - |
| **Cohort - EDEN** | 13.2% (1.6%, 26%) | 0.025 | 12.4% (0.78%, 25%) | 0.036 |
| **Cohort - INMA** | 7.89% (0.84%, 15%) | 0.028 | 8.12% (0.96%, 16%) | 0.026 |
| **Cohort - KANC** | 4.5% (-0.36%, 9.6%) | 0.07 | 5.18% (-0.15%, 11%) | 0.057 |
| **Cohort - MOBA** | 7.16% (0.57%, 14%) | 0.033 | 5.92% (-0.75%, 13%) | 0.083 |
| **Cohort - RHEA** | 6.62% (1.9%, 12%) | 0.0059 | 7.6% (2.2%, 13%) | 0.0052 |
| **Ethnicity - White** | - | - | - | - |
| **Ethnicity - Pakistani or other Asian** | 7.15% (1.9%, 13%) | 0.0071 | 8.12% (2.6%, 14%) | 0.0034 |
| **Ethnicity - Other** | 6.67% (-1%, 15%) | 0.091 | 7.21% (-0.59%, 16%) | 0.071 |
| **Parental Education - Low** | - | - | - | - |
| **Parental Education - Middle** | -0.37% (-4.7%, 4.2%) | 0.87 | -0.271% (-4.6%, 4.3%) | 0.9 |
| **Parental Education - High** | -0.146% (-4.7%, 4.6%) | 0.95 | -0.42% (-5%, 4.4%) | 0.86 |
| **Family Social Capital - Low** | - | - | - | - |
| **Family Social Capital - Medium** | 1.32% (-1.4%, 4.1%) | 0.35 | 1.21% (-1.6%, 4.1%) | 0.4 |
| **Family Social Capital - High** | 2.1% (-0.69%, 5%) | 0.14 | 2.15% (-0.72%, 5.1%) | 0.14 |
| **Birth weight (kg)** |  |  | 1.77% (-0.91%, 4.5%) | 0.2 |
| **Gestational age (weeks)** |  |  | 0.00647% (-0.77%, 0.79%) | 0.99 |
| **Prenatal smoke exposure - None** |  |  | - | - |
| **Prenatal smoke exposure -Passive** |  |  | -1.15% (-3.9%, 1.7%) | 0.42 |
| **Prenatal smoke exposure - Active** |  |  | -0.589% (-4%, 3%) | 0.74 |
| **Moderate-to-Vigorous Physical Activity (min/day)** |  |  | -0.0218% (-0.049%, 0.0059%) | 0.12 |
| **Sedentary Behaviour** |  |  | 0.00237% (-0.0066%, 0.011%) | 0.6 |
| **KIDMED Diet Score** |  |  | 0.394% (-0.25%, 1%) | 0.23 |
| **Fat mass percentage of total weight (%)** |  |  | -0.144% (-0.3%, 0.01%) | 0.068 |
| **Height (m)** |  |  | -4.44% (-22%, 17%) | 0.66 |
| **Environmental tobacco smoke exposure - No** |  |  | - | - |
| **Environmental tobacco smoke exposure - Yes** |  |  | 0.013% (-2.4%, 2.5%) | 0.99 |
| **Maternal Perceived Stress Score** |  |  | 0.0474% (-0.34%, 0.44%) | 0.81 |
| **NK** | -0.322% (-0.6%, -0.045%) | 0.023 | -0.302% (-0.58%, -0.023%) | 0.034 |
| **Bcell** | 0.15% (-0.17%, 0.47%) | 0.35 | 0.16% (-0.16%, 0.48%) | 0.32 |
| **CD4T** | 0.243% (0.039%, 0.45%) | 0.02 | 0.218% (0.012%, 0.42%) | 0.038 |
| **CD8T** | 0.206% (-0.055%, 0.47%) | 0.12 | 0.206% (-0.057%, 0.47%) | 0.12 |
| **Eos** | -0.174% (-0.89%, 0.55%) | 0.64 | -0.126% (-0.85%, 0.6%) | 0.73 |
| **Mono** | 0.092% (-0.32%, 0.51%) | 0.66 | 0.111% (-0.31%, 0.53%) | 0.6 |

**Table S2 Sequentially adjusted models examining the relationship between cortisol production and Telomere length (n = 957). Each model is adjusted for all covariates shown in respective columns.**

| **Telomere length** | **Model 1 (Basic model)** | | **Model 2 (Total and direct effect)** | | **Model 3 (Total and direct effect plus adjustment for immune cell proportions)** | |
| --- | --- | --- | --- | --- | --- | --- |
|  | **% changes (95% CI)** | **P-value** | **% changes (95% CI)** | **P-value** | **% changes (95% CI)** | **P-value** |
| **Cortisol production (µg /µmol creatinine)** | -0.398% (-3.1%, 2.4%) | 0.78 | 0.325% (-2.5%, 3.2%) | 0.82 | 0.566% (-2.2%, 3.4%) | 0.69 |
| **Sex - Male** | - | - | - | - | - | - |
| **Sex - Female** | 4.55% (2.1%, 7%) | <0.0001 | 5.08% (2.6%, 7.7%) | <0.0001 | 5.13% (2.6%, 7.7%) | <0.0001 |
| **Age (years)** | -1.65% (-4.1%, 0.84%) | 0.19 | -1.52% (-4%, 1%) | 0.23 | -1.72% (-4.2%, 0.79%) | 0.18 |
| **Cohort - BIB** | - | - | - | - | - | - |
| **Cohort - EDEN** | 8.26% (-3.9%, 22%) | 0.19 | 7.02% (-5.3%, 21%) | 0.28 | 10.3% (-2.3%, 25%) | 0.11 |
| **Cohort - INMA** | 4.21% (-3.6%, 13%) | 0.3 | 4.36% (-3.6%, 13%) | 0.29 | 6.57% (-1.5%, 15%) | 0.11 |
| **Cohort - KANC** | 1.24% (-4.1%, 6.9%) | 0.66 | 2.43% (-3.5%, 8.8%) | 0.43 | 4.54% (-1.7%, 11%) | 0.15 |
| **Cohort - MOBA** | 6.88% (-0.49%, 15%) | 0.068 | 4.51% (-3.1%, 13%) | 0.25 | 5.11% (-2.5%, 13%) | 0.19 |
| **Cohort - RHEA** | 5.85% (0.044%, 12%) | 0.048 | 5.1% (-1.1%, 12%) | 0.11 | 4.09% (-2%, 11%) | 0.19 |
| **Ethnicity - White** | - | - | - | - | - | - |
| **Ethnicity - Pakistani or other Asian** | 7.39% (0.96%, 14%) | 0.024 | 8.93% (2.3%, 16%) | 0.0077 | 10.1% (3.4%, 17%) | 0.0028 |
| **Ethnicity - Other** | 8.52% (-0.59%, 18%) | 0.068 | 9.56% (0.27%, 20%) | 0.043 | 9.01% (-0.19%, 19%) | 0.055 |
| **Parental Education - Low** |  |  | - | - | - | - |
| **Parental Education - Middle** |  |  | -1.75% (-6.9%, 3.7%) | 0.52 | -1.09% (-6.2%, 4.3%) | 0.69 |
| **Parental Education - High** |  |  | -1.95% (-7.4%, 3.8%) | 0.5 | -1.27% (-6.7%, 4.5%) | 0.66 |
| **Family Social Capital - Low** |  |  | - | - | - | - |
| **Family Social Capital - Medium** |  |  | -0.137% (-3.3%, 3.1%) | 0.93 | 0.356% (-2.8%, 3.6%) | 0.83 |
| **Family Social Capital - High** |  |  | 1.49% (-1.7%, 4.8%) | 0.37 | 1.38% (-1.8%, 4.7%) | 0.4 |
| **Family Affluence Scale - Low** |  |  | - | - | - | - |
| **Family Affluence Scale - Medium** |  |  | 2.45% (-2.1%, 7.2%) | 0.3 | 1.89% (-2.6%, 6.6%) | 0.42 |
| **Family Affluence Scale - High** |  |  | 4.66% (-0.18%, 9.7%) | 0.059 | 3.74% (-1%, 8.7%) | 0.13 |
| **Moderate-to-Vigorous Physical Activity (min/day)** |  |  | -0.0283% (-0.059%, 0.0025%) | 0.072 | -0.0283% (-0.059%, 0.0025%) | 0.071 |
| **Sedentary Behaviour** |  |  | 0.00404% (-0.0061%, 0.014%) | 0.43 | 0.00298% (-0.0071%, 0.013%) | 0.56 |
| **KIDMED Diet Score** |  |  | 0.382% (-0.34%, 1.1%) | 0.3 | 0.387% (-0.33%, 1.1%) | 0.29 |
| **Fat mass percentage of total weight (%)** |  |  | -0.238% (-0.41%, -0.071%) | 0.0053 | -0.208% (-0.38%, -0.04%) | 0.015 |
| **Environmental tobacco smoke exposure - No** |  |  | - | - | - | - |
| **Environmental tobacco smoke exposure - Yes** |  |  | 0.937% (-1.8%, 3.7%) | 0.5 | 1.25% (-1.4%, 4%) | 0.37 |
| **Maternal Perceived Stress Score** |  |  | -0.0393% (-0.49%, 0.41%) | 0.86 | -0.0238% (-0.47%, 0.42%) | 0.92 |
| **NK** |  |  |  |  | -0.263% (-0.56%, 0.038%) | 0.087 |
| **Bcell** |  |  |  |  | 0.218% (-0.15%, 0.58%) | 0.24 |
| **CD4T** |  |  |  |  | 0.15% (-0.083%, 0.38%) | 0.21 |
| **CD8T** |  |  |  |  | 0.354% (0.064%, 0.65%) | 0.017 |
| **Eos** |  |  |  |  | -0.273% (-1.1%, 0.51%) | 0.49 |
| **Mono** |  |  |  |  | 0.188% (-0.28%, 0.66%) | 0.43 |

**Figure S2: Summary of mediation analysis of potential mediators of the association of family affluence scale (FAS) and telomere length (TL). Error bars show 95% confidence intervals (n = 1160).** Covariates included in the mediation analysis: age, sex, cohort, ethnicity, family social capital, parental education, birthweight, gestational age, smoking during pregnancy, fat mass, physical activity, KIDMED score, height, environmental tobacco smoke exposure, maternal perceived stress. Additionally, natural killer, CD4+T, CD8+T cells and cortisol production were included as covariates when testing biomarkers as potential mediators


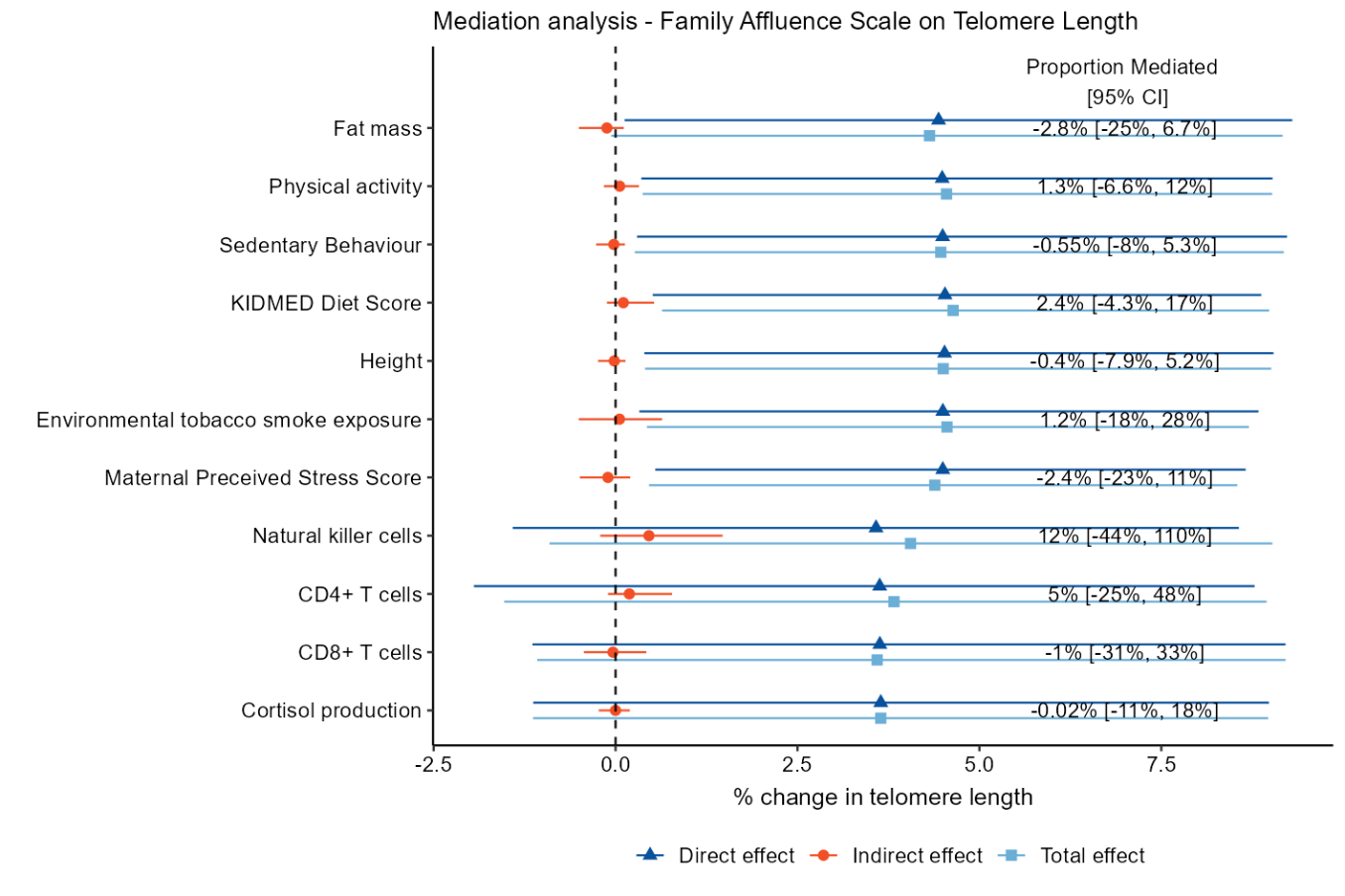


**Supplementary acknowledgement:**

The authors thank the EDEN mother-child study group, whose members are: I. Annesi-Maesano, J.Y. Bernard, J. Botton, M.A. Charles, P. Dargent-Molina, B. de Lauzon-Guillain, P. Ducimetière, M. de Agostini, B. Foliguet, A. Forhan, X. Fritel, A. Germa, V. Goua, R. Hankard, B. Heude, M. Kaminski, B. Larroque†, N. Lelong, J. Lepeule, G. Magnin, L. Marchand, C. Nabet, F Pierre, R. Slama, M.J. Saurel-Cubizolles, M. Schweitzer, O. Thiebaugeorges.
